# Phantom and clinical assessment of small pulmonary nodules using Q.Clear reconstruction on a silicon-photomultiplier-based time-of-flight PET/CT system

**DOI:** 10.1038/s41598-021-89725-z

**Published:** 2021-05-14

**Authors:** Zhifang Wu, Binwei Guo, Bin Huang, Xinzhong Hao, Ping Wu, Bin Zhao, Zhixing Qin, Jun Xie, Sijin Li

**Affiliations:** 1 Department of Nuclear Medicine, First Hospital of Shanxi Medical University, No. 85 South Jiefang Road, Taiyuan, 030001 Shanxi People’s Republic of China; 2 Molecular Imaging Precision Medical Collaborative Innovation Center, Shanxi Medical University, Taiyuan, Shanxi People’s Republic of China; 3 Department of Biochemistry and Molecular Biology, Shanxi Medical University, Taiyuan, Shanxi People’s Republic of China

**Keywords:** Cancer, Medical research, Physics

## Abstract

To evaluate the quantification accuracy of different positron emission tomography-computed tomography (PET/CT) reconstruction algorithms, we measured the recovery coefficient (RC) and contrast recovery (CR) in phantom studies. The results played a guiding role in the partial-volume-effect correction (PVC) for following clinical evaluations. The PET images were reconstructed with four different methods: ordered subsets expectation maximization (OSEM), OSEM with time-of-flight (TOF), OSEM with TOF and point spread function (PSF), and Bayesian penalized likelihood (BPL, known as Q.Clear in the PET/CT of GE Healthcare). In clinical studies, SUVmax and SUVmean (the maximum and mean of the standardized uptake values, SUVs) of 75 small pulmonary nodules (sub-centimeter group: < 10 mm and medium-size group: 10–25 mm) were measured from 26 patients. Results show that Q.Clear produced higher RC and CR values, which can improve quantification accuracy compared with other methods (P < 0.05), except for the RC of 37 mm sphere (P > 0.05). The SUVs of sub-centimeter fludeoxyglucose (FDG)-avid pulmonary nodules with Q.Clear illustrated highly significant differences from those reconstructed with other algorithms (P < 0.001). After performing the PVC, highly significant differences (P < 0.001) still existed in the SUVmean measured by Q.Clear comparing with those measured by the other algorithms. Our results suggest that the Q.Clear reconstruction algorithm improved the quantification accuracy towards the true uptake, which potentially promotes the diagnostic confidence and treatment response evaluations with PET/CT imaging, especially for the sub-centimeter pulmonary nodules. For small lesions, PVC is essential.

## Introduction

Quantitative PET imaging shows growing importance in the early-stage disease diagnosis^1,2^ and the treatment response evaluation^3,4^ by noninvasively monitoring the physiological and pathological processes in vivo. In the development of PET imaging systems, many research efforts have been devoted to reinforcing the accuracy of quantifications and the detectability of small lesions^5,6^. Due to the partial volume effect caused by the limited spatial resolution of conventional PET systems, the radiotracer uptake is usually underestimated when lesions are three times smaller than the spatial resolution^7^. The traditional iterative image reconstruction algorithm, e.g., OSEM, is not able to reach full convergence by increasing the number of iterations because the noise grows with more iterations. There is a compromise between the number of iterations and noise^8,9^. Therefore, it is particularly difficult to assess the true metabolic activity of small lesions, such as sub-centimeter pulmonary nodules or lymph nodes. Many studies demonstrated the challenges of evaluating sub-centimeter nodules. For instance, the differential diagnostic sensitivity became lower for determining malignancy of such nodules than that of larger ones^10–12^, and even false-negative findings can be generated^13^. The visibility of small lesions and quantification accuracy facilitates improved staging, treatment planning, response monitoring, and prognostic estimation, which are important for clinical diagnostic confidence and patient management^14^.

Generally, it is impossible to know the true uptake of a lesion in vivo. In order to evaluate the quantification accuracy, the most reliable method is to measure the recovery coefficient (RC) in the phantom study, which gives the true activity by calculating the ratio of measured activity to the true one determined by the dose calibrator^15^. The contrast recovery (CR) is another important index reflecting the true uptake ratio in the lesion and background by showing the ratio between measured sphere-to-background activity and designed sphere-to-background activity^16^. The BPL-based reconstruction algorithm (known as Q.Clear in the PET/CT of GE Healthcare) on a digital PET/CT system (Discovery MI, GE Healthcare) has shown significant advantages over the conventional reconstruction algorithm (OSEM) in photomultiplier tube (PMT)-based PET/CT scanners^17–20^. It has been revealed that the new Q.Clear PET images provided significant increases in signal-to-background, signal-to-noise (SNR) ratios, and SUVs, with greatly enhanced visual sensitivity for assessing small pulmonary nodules^21,22^, liver metastasis^23^, and mediastinal nodes in non-small cell lung cancer^24^. Although SUVs measured from the Q.Clear achieved higher values, it needs to be further verified that this SUV elevation reflects higher quantification accuracy.

Therefore, we evaluated the quantification accuracy of Q.Clear reconstructions on a SiPM-based PET/CT system with a phantom study and performed a clinical study on pulmonary nodules based on the results of the phantom study.

## Results

### Phantom

**Figure 1:**
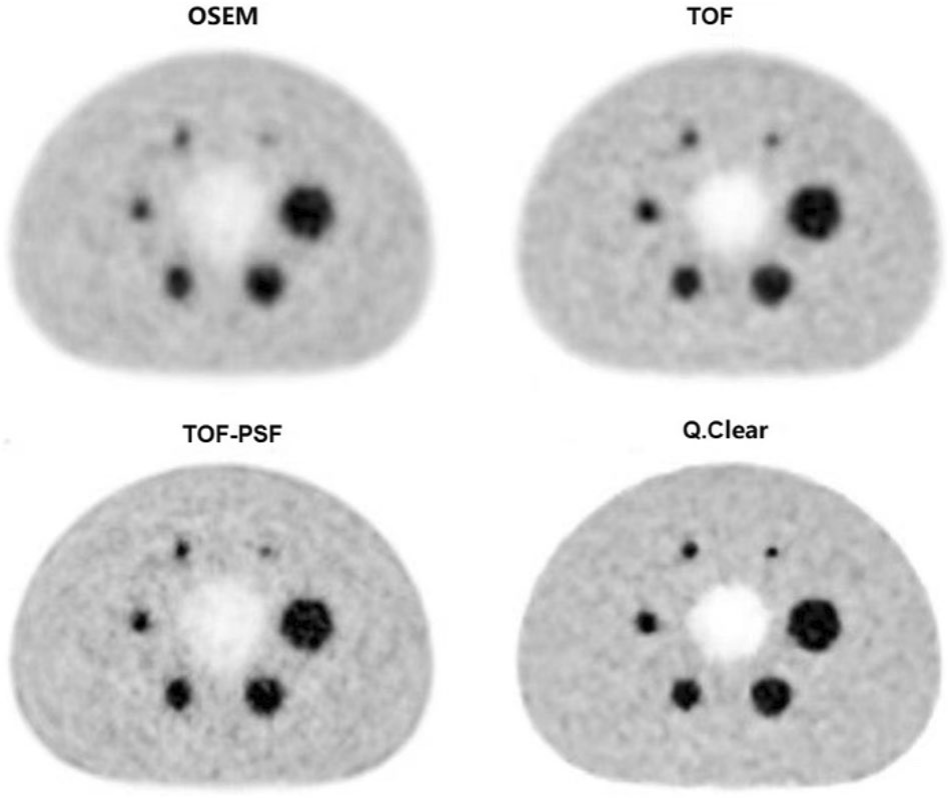
Comparison of PET reconstruction methods for the NEMA phantom with 6 spheres (diameter 10–37 mm) filled with 13.2 kBq/mL Fluoride ions in a 4-to-1 contrast ratio. The mean uptake values (Bq/mL) of the 6 spheres (in order from 10 to 37 mm) for 4 reconstructions were as follow: 4.12, 5.66, 6.57, 7.52, 9.03, 9.78 for OSEM, 5.36, 7.25, 8.32, 8.77, 9.62, 10.24 for TOF, 5.76, 7.86, 8.84, 8.96, 9.67, 10.47 for TOF-PSF, 6.70, 9.09, 9.54, 9.62, 10.99, 11.15 for Q.Clear. All the PET images are displayed on SUV scale 0–8.

From the visual assessment (Fig. 1), as more advanced reconstruction techniques (from OSEM to Q.Clear reconstruction) were introduced into the PET reconstruction, the overall image quality improved. Thanks to the Q.Clear algorithm, the smallest sphere (diameter = 10 mm) was particularly outstanding with clearer boundaries; meanwhile, reduced background noise was also achieved compared with the other reconstruction methods.

**Figure 2:**
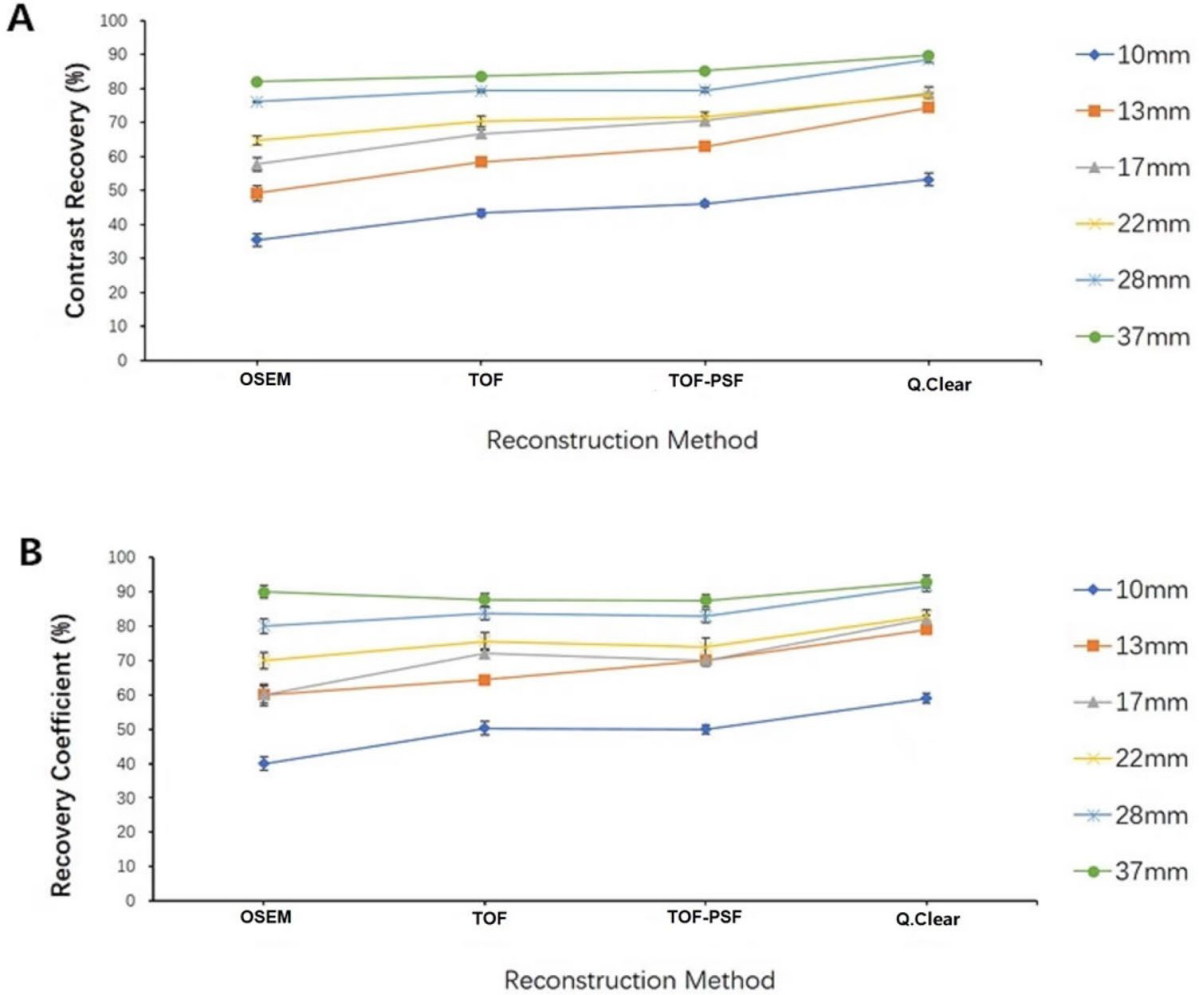
Comparison of recovery coefficient (**A**) and contrast recovery (**B**) among PET reconstruction methods for 6 hot spheres (diameter 10–37 mm) filled with 13.2 kBq/mL Fluoride ions in a 4-to-1 contrast ratio.

Table 1 and Fig. 2 has shown that A_m_ (Bq/mL), CR (%), and RC (%) increased gradually in the order from OSEM, TOF, TOF-PSF to Q.Clear. The average A_m_ and RC of 5 spheres (10 mm–28 mm) using the Q.Clear algorithm were significantly higher than those of other reconstruction methods (P < 0.05), while the biggest sphere (37 mm) showed no significant difference among reconstruction methods (P > 0.05). The CR values of all hot spheres using the Q.Clear algorithm had higher values than those of the other reconstruction algorithms (P < 0.05) (Table 2). The RC values of 6 hot spheres calculated from the Q.Clear algorithm reached up to 59.0% (10 mm), 79.0% (13 mm), 82.0% (17 mm), 83.0% (22 mm), 91.7% (28 mm) and 92.9% (37 mm) while CR values reached up to 53.2% (10 mm), 74.4% (13 mm), 78.7% (17 mm), 78.0% (22 mm), 88.5% (28 mm) and 89.8% (37 mm).

**Table 1:** The average uptake Am, recovery coefficient, and contrast recovery determined from 6 hot spheres.

Sphere	OSEM			TOF		
	A_m_ (Bq/mL)	RC (%)	CR (%)	A_m_ (Bq/mL)	RC (%)	CR (%)
10 mm	4.34 ± 0.25	40.0 ± 1.9	35.5 ± 1.88	5.31 ± 0.25	50.3 ± 1.90	43.4 ± 1.17
13 mm	6.00 ± 0.34	60.0 ± 2.5	49.2 ± 2.26	7.15 ± 0.10	64.3 ± 0.70	58.4 ± 1.20
17 mm	7.04 ± 0.42	60.0 ± 3.2	57.7 ± 2.00	8.16 ± 0.17	72.0 ± 1.30	66.7 ± 1.33
22 mm	7.89 ± 0.32	70.0 ± 2.4	64.7 ± 1.32	8.62 ± 0.34	75.5 ± 2.60	70.4 ± 1.60
28 mm	9.29 ± 0.26	80.0 ± 2.0	76.1 ± 0.19	9.70 ± 0.24	83.7 ± 1.80	79.3 ± 0.49
37 mm	10.00 ± 0.24	90.0 ± 1.8	82.0 ± 0.40	10.22 ± 0.25	87.7 ± 1.90	83.6 ± 0.34

	TOF-PSF			Q.Clear		
	A_m_ (Bq/mL)	RC (%)	CR (%)	A_m_ (Bq/mL)	RC (%)	CR (%)
10 mm	5.66 ± 0.17	50.0 ± 1.30	46.1 ± 0.70	6.80 ± 0.45	59.0 ± 1.40	53.2 ± 1.89
13 mm	7.73 ± 0.16	70.0 ± 1.20	63.0 ± 1.04	9.04 ± 0.13	79.0 ± 1.00	74.4 ± 0.59
17 mm	8.69 ± 0.19	70.0 ± 1.50	70.6 ± 1.32	9.57 ± 0.42	82.0 ± 1.20	78.7 ± 1.77
22 mm	8.80 ± 0.34	73.9 ± 2.60	71.7 ± 1.43	9.49 ± 0.21	83.0 ± 1.60	78.0 ± 0.54
28 mm	9.76 ± 0.23	82.8 ± 1.80	79.5 ± 0.66	10.76 ± 0.21	91.7 ± 1.60	88.5 ± 0.78
37 mm	10.45 ± 0.26	87.4 ± 1.90	85.2 ± 0.15	10.92 ± 0.24	92.9 ± 1.80	89.8 ± 0.52

**Table 2:** The significant difference in 6 spheres among 4 reconstruction methods (P value).

Parameters	Sphere					
	10 mm	13 mm	17 mm	22 mm	28 mm	37 mm
A_m_	0.018	0.016	0.016	0.022	0.031	0.064
CR	0.016	0.016	0.016	0.022	0.023	0.016
RC	0.018	0.016	0.016	0.022	0.031	0.075

All P values were generated from Kruskal Wallis H test among the four reconstruction algorithms.

### Clinical characteristics

**Figure 3:**
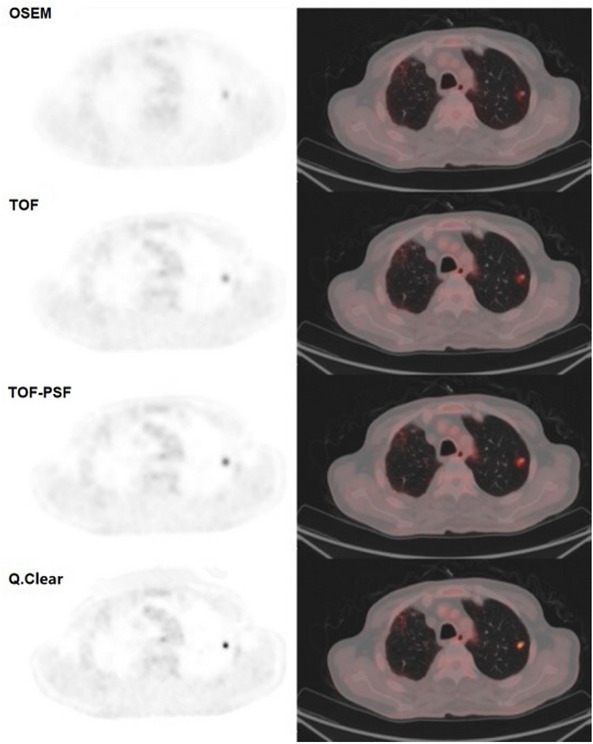
Illustration of PET images of a pulmonary nodule (long-axis diameter 7.2 mm) reconstructed with different algorithms. The SUVmax of OSEM, TOF, TOF-PSF, Q.Clear reconstructed images were 1.93, 2.62, 3.9, 7.28 respectively. The SUVmean were 0.99, 1.42, 1.87, 3.46 respectively. All the PET images are displayed on SUV scale 0–8.

**Figure 4:**
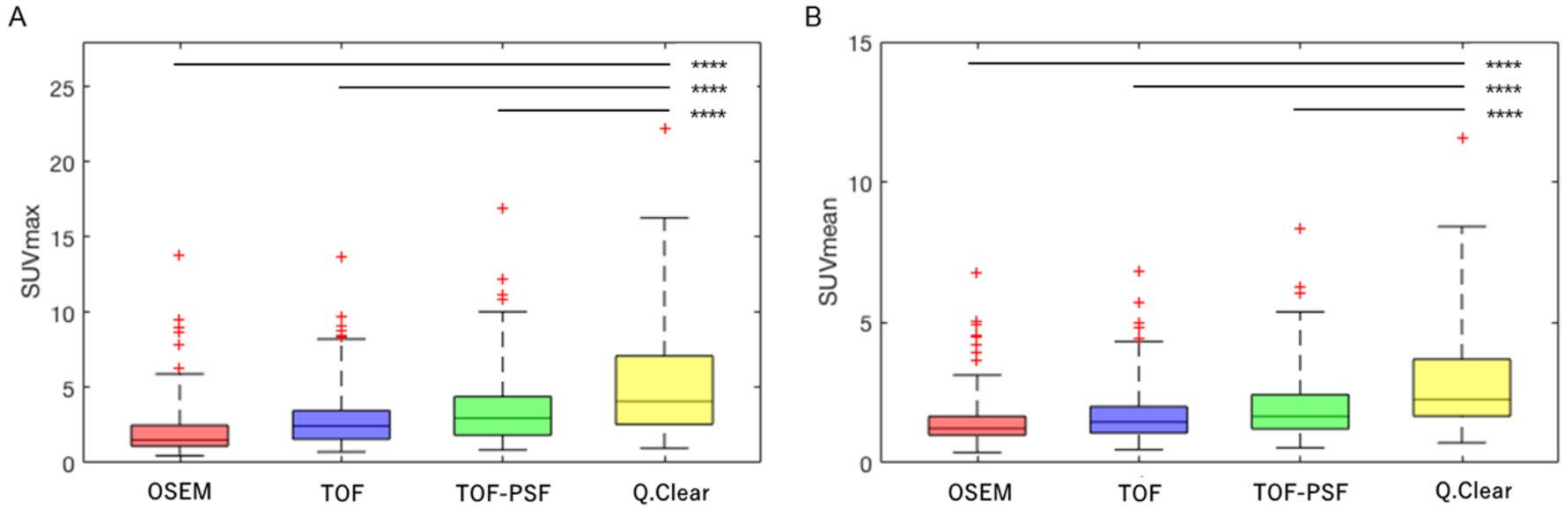
The box plots of (**A**) SUVmax and (**B**) SUVmean measurements of 75 pulmonary nodules on PET images reconstructed with different methods. Highly statistically significant differences (noted as ****P values < 0.001) in the SUVmax and SUVmean were shown between PET images reconstructed with Q.Clear and each of the other 3 methods (OSEM, TOF, and TOF-PSF).

An illustration of the same pulmonary nodule (long-axis diameter 7.2 mm) on the PET images reconstructed using different reconstruction algorithms was shown in Fig. 3. The lesion became clearer when the Q.Clear reconstruction was used. The SUVmax and SUVmean calculated from all the small pulmonary nodules (n = 75) increased gradually in the order from OSEM, TOF, TOF-PSF to Q.Clear and SUVs determined from the Q.Clear algorithm were significantly higher than those from other reconstruction methods (P < 0.001 for SUVmax and SUVmean) (Fig. 4).

However, the significance was highly affected by the size of nodules, as shown in Table 3. SUVmax and SUVmean determined from sub-centimeter pulmonary nodules had highly significant differences among different reconstruction techniques (P < 0.001), while the medium-size group showed no significant difference (P > 0.05).

**Table 3:** The impact of different reconstruction methods on SUVs of small pulmonary nodules before/after PVC.

Groups	Size (mm)	SUVmax					SUVmean				
		OSEM	TOF	TOF-PSF	Q.Clear	P value	OSEM	TOF	TOF-PSF	Q.Clear	P value
** ≤ 10 mm**											
n = 57	5.03 ± 1.79	1.58 ± 0.82	2.26 ± 1.07	2.76 ± 1.39	4.32 ± 2.46	0.000	1.18 ± 0.53	1.45 ± 0.69	1.63 ± 0.76	2.50 ± 1.32	0.000
**10–25 mm**											
n = 18	15.12 ± 4.32	5.52 ± 3.56	6.13 ± 3.44	7.23 ± 4.24	8.77 ± 5.65	0.128	3.04 ± 1.70	3.31 ± 1.68	3.76 ± 2.00	4.92 ± 2.90	0.050

Groups	Size (mm)	Corr SUVmax					Corr SUVmean				
		OSEM	TOF	TOF-PSF	Q.Clear	P value	OSEM	TOF	TOF-PSF	Q.Clear	P value
** ≤ 10 mm**											
n = 57	5.03 ± 1.79	6.78 ± 3.01	6.66 ± 2.16	7.17 ± 2.78	8.64 ± 4.47	0.004	4.24 ± 2.44	4.33 ± 1.54	4.27 ± 1.54	5.04 ± 2.56	0.024
**10–25 mm**											
n = 18	15.12 ± 4.32	9.36 ± 5.99	9.51 ± 5.64	11.04 ± 6.90	11.75 ± 8.03	0.649	5.25 ± 3.05	5.16 ± 2.85	5.76 ± 3.29	6.60 ± 4.14	0.559

All P values were generated from Kruskal Wallis H test among the four reconstruction algorithms.

### PVC for pulmonary nodules

**Figure 5:**
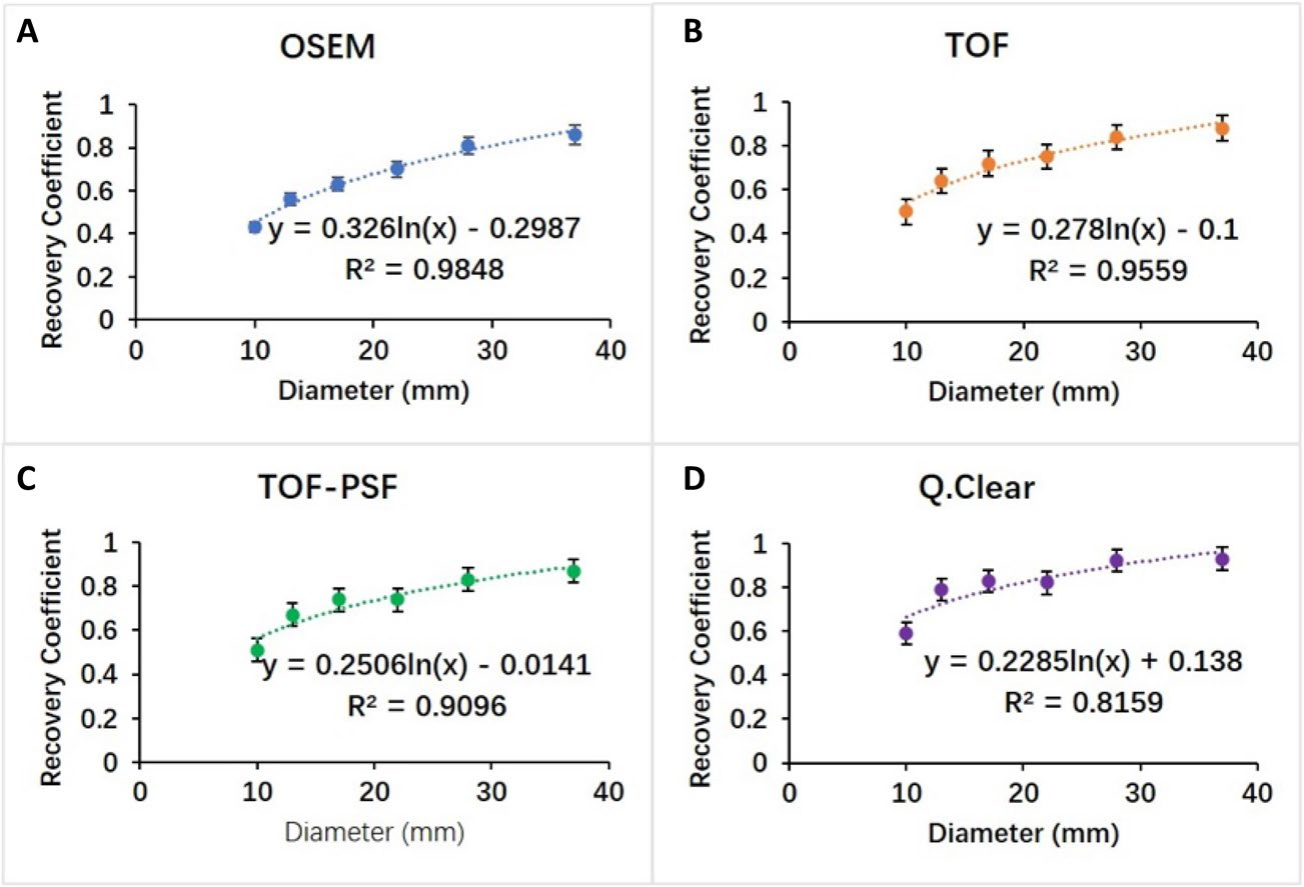
Recovery coefficients calculated based on PET images reconstructed with different algorithms (**A**) OSEM, (**B**) TOF, (**C**) TOF-PSF, (**D**) Q.Clear, showing as a function of measured sphere diameter on CT images. The equations and R2 values of linear fittings between recovery coefficient and measured sphere diameter were presented. Error bars shown are 1 SD.

**Figure 6:**
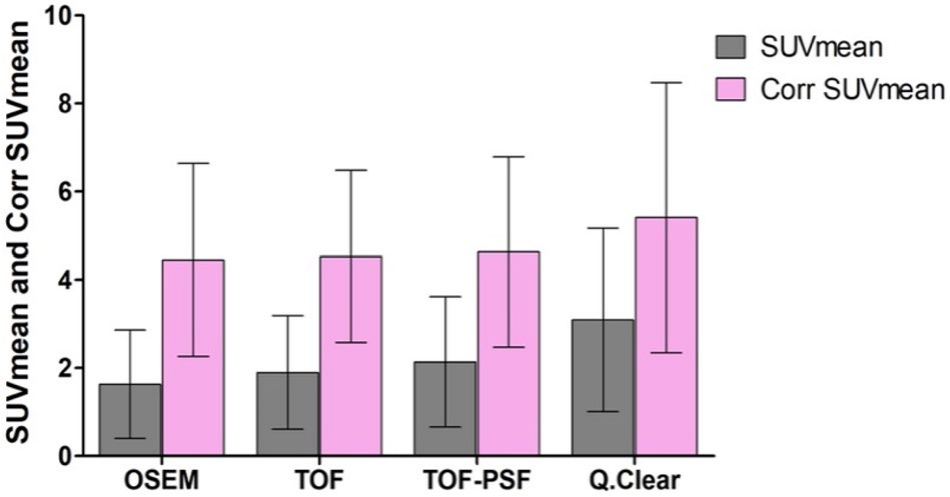
The histogram plots of SUVmean and PVE corrected SUVmean (corr SUVmean) of 75 pulmonary nodules on PET images reconstructed with different methods. After the PVE correction, the SUVmean increases represented by %△SUVmean were 160.1%, 138.4%, 116.4%, and 75.1% for OSEM, TOF, TOF-PSF and Q.Clear, respectively.

In this study, we also performed the RC linear regression from phantom studies (Fig. 5) to apply PVC on SUVs of all pulmonary nodules. The original SUVmean and the PVE corrected SUVmean (corr SUVmean) measured from 4 reconstruction methods were shown in Fig. 6.

The PVC %ΔSUVmeans were 160.1%, 138.4%, 116.4%, and 75.1% for OSEM, TOF, TOF-PSF and Q.Clear, respectively (P < 0.001). A highly significant difference existed not only between the original SUVmean and the corr SUVmean in each reconstruction algorithm (P < 0.001 for all) but also between the Q.Clear algorithm and the other three ones in corr SUVmean (P < 0.05 for all). However, differences in SUVs measured from four reconstruction methods were not significant for the medium-size group (Table 3, P = 0.649 for SUVmax and P = 0.559 for SUVmean).

## Discussion

A reliable and precise measurement of radiopharmaceutical uptake is more and more important in PET, particularly for differential diagnosis, treatment planning, and therapy response evaluation. It is still challenging to achieve accurate quantification of radiotracer uptake in the small lesions due to the partial convergence in current reconstruction algorithms and the partial volume effect, which usually underestimate SUV. In this study, we demonstrated the values of using the Q.Clear reconstruction algorithm on the SiPM-based PET/CT platform for improving quantification accuracy and image quality. The Q.Clear reconstruction algorithm revealed considerable enhancement towards true uptake compared with the other reconstruction methods, especially in sub-centimeter pulmonary nodules. In this study, image reconstruction parameters were chosen based on the routine clinical protocols in our hospital. Small lesions are more clinically challenging. It is hard to reach full convergence on the routine OSEM based algorithms and is heavily affected by PVE. Nodules with a size under 25 mm were considered as small lesions.

The Q.Clear-reconstructed images showed the highest RC and CR compared to the other three methods (Fig. 2), suggesting the Q.Clear reconstruction algorithm improved the quantification accuracy of PET imaging approaching the true uptake. However, it was found that the RC difference among the four reconstruction methods was not significant (P > 0.05) in the largest sphere (37 mm). The reason for this is, under the same number of iterations, the degree of convergence of OSEM based reconstruction algorithms was higher in large subjects, which can be seen in Fig. 2 that larger spheres always had higher RC values compared with smaller spheres regardless of reconstruction methods used.

For the quantification of pulmonary nodules, significant increases in the SUVmax and SUVmean were observed when reconstructed with the Q.Clear algorithm compared to other three methods (Fig. 4), and the increases in SUVs for those of sub-centimeter nodules were significantly greater than those of the medium-size group (Table 3, P < 0.001). By comparing the changes of SUVs among different reconstruction methods in the medium-size group, increments can be found but were not statistically significant (Table 3, P > 0.05). This was because, firstly, the medium-size group was closer to full convergence on routine OSEM based algorithms compared to the sub-centimeter group; secondly, the number of nodules enrolled in the medium-size group was inadequate (n = 18) to reach statistical significance. Similarly, Teoh et al. also showed that the greatest improvement was found in malignancy detection in nodules ≤ 10 mm compared with larger ones^21^. This concluded the Q.Clear algorithm exerted more profound impacts on the SUV measurement of small pulmonary nodules, which potentially facilitated the enhancement of the lesion visibility and detectability, especially for sub-centimeter lesions.

The partial volume effect is another impacting factor on SUV quantification accuracy of small lesions, which also underestimates the true uptake. In our phantom study, RC was calculated to reveal the direct relationship between the measured and true radioactivity. Then the association between RC values and sphere diameter measured on CT images was established for each reconstruction method. The linear regression was performed, and good regression coefficients were achieved (Fig. 5). Based on the equations and the measured nodule size in CT images, we corrected the partial volume effect to improve the SUV measurement accuracy of all pulmonary nodules. This RC-based PVE correction approach is on assumptions that the lesion has a regular spherical shape and uniform distribution of radioactivity^25^. Due to the limited size range of phantom spheres being from 10 to 37 mm, the PVE correction for those nodules either smaller than 10 mm or larger than 37 mm should be careful. In addition, in Fig. 6, the number of outlier values decreased in the order from OSEM, TOF, TOF + PSF to Q.Clear, which indicated the improvement in the SUV reliability. A few previous studies have demonstrated this method for PVE correction^11,26,27^. For instance, Srinivas et al. created a ‘lookup table’ for determining approximate RC values for lesions in different sizes and disease regions^25^. Our finding on the relationship between RC and sphere diameter showed consistency with their result. Future elaborated phantom experiment designs should be dedicated to investigating different clinical imaging scenarios with different lesion sizes, lesion shapes, image contrasts, and radioactivity levels. It should be noted that this PVE correction method is equipment-specific, which means different PET scanners may have different RC-diameter behaviors.

The respiratory motion during PET imaging may compromise the spatial resolution, resulting in smearing or blurring effects of PET images, which would eventually affect SUV measurement. Existing researches suggested that respiratory movement leads to overestimations of radiotracer-avid target volume and reduction in the SUV due to the recorded number of coincidence events tending to distribute in a larger volume^28^. A data-driven respiratory gating function is now available on the GE system, which has shown out-performance than the device-based gating^29^. The respiratory gating system can be employed in a future study to facilitate the improvement of spatial resolution and quantification accuracy, especially when radiotherapy treatment planning is desired^30–32^.

Furthermore, the small pulmonary nodules in our study were not histopathologically verified. The relationship between malignancy and SUV measurement of pulmonary nodules under Q.Clear reconstruction need to be investigated, which could benefit differential diagnosis based on quantitative PET/CT imaging.

## Conclusion

In our phantom and clinical studies, the Q.Clear reconstruction algorithm combined with SiPM-based digital PET/CT platform significantly improved the quantification accuracy towards the true uptake by accessing RC and CR values, which potentially promotes the diagnostic confidence and treatment response evaluation with PET/CT imaging, especially for the sub-centimeter pulmonary nodules. For small lesions, the PVE correction is essential.

## Methods and materials

### Phantom

A National Electrical Manufacturers Association (NEMA) image quality phantom^33^ was utilized in this study with six spheres filled with 13.2 kBq/mL Fluoride ions in a 4-to-1 ratio (sphere to background activity concentration). Recovery coefficient (RC) and contrast recovery (CR) were measured using the following equations (Eqs. 1, 2):

1 $$RC=\frac{{A}_{M}}{{A}_{K}}\times 100\%$$

2 $$CR=\frac{\frac{{A}_{M}}{{A}_{B}}-1}{C-1}\times 100\%$$

where *A*_*M*_ is the measured activity (in kBq/mL) in each sphere delineated on CT images; A_*B*_ is the measured activity in the background. *A*_*K*_ is the known activity (in kBq/mL) in the sphere; *C* is the known ratio of activity in the sphere to the background (that is 4:1 in the study).

### PET/CT imaging

^18^F-FDG PET/CT scans were performed on a SiPM-based digital PET/CT system (Discovery MI, GE Healthcare, USA) equipped with Q.Clear (GE Healthcare) reconstruction algorithm with an axial field of view 25 mm. Before scanning, all the patients fasted for at least 6 h with blood glucose level being lower than 200 mg/dL. Then, the patients received 2.96–3.70 MBq/kg of ^18^F-FDG and rested for approximately 60 min after injection. The CT images were acquired using a slice thickness of 3.75 mm, a pitch of 0.984, 120 kVp, 60–150 mA modulation with a noise index of 18, rotation time of 0.5 s. PET imaging was conducted with 3 min/bed in List-mode that allows for post-processing PET image reconstructions. The phantom was scanned three times with the same PET/CT imaging parameters.

### Image reconstruction

All the phantom and clinical PET data were reconstructed using four different algorithms, namely OSEM (2 iterations, 17 subsets, 6.4-mm post-filter cutoff), TOF: OSEM + TOF (time-of-flight, with same OSEM parameters except for 3 iterations), TOF-PSF: OSEM + TOF + PSF (point-spread-function, same OSEM parameters except for 3 iterations), Q.Clear: TOF + PSF + BPL (β = 350), where β is defined in the Q.Clear objective function as below,

3 $$\widehat{x}=\mathrm{arg}{max}_{x\ge 0}\sum {y}_{i}\mathrm{log}{[{P}_{x}]}_{i}-{\left[{P}_{x}\right]}_{i}-\beta R(x)$$

where *x* is the image estimate; *i* is the pixel index; *y*_*i*_ represents the measured PET coincidence data; *P* is the system geometry matrix; β is the penalization factor; R(x) is the penalty to control noise. After a large number of experiments in our department, 350 is the best beta setting, which is used in our clinical practice.

### Clinical evaluation

The clinical study had been approved by the Ethics Committee of First Hospital of Shanxi Medical University (Ethical Review Number K005). All methods of this experiment are implemented in accordance with the guidelines and regulations related to GCP and ICH-GCP. This clinical study is a retrospective study. It only collects clinical data of patients, does not interfere with the treatment plan of patients, and will not bring risks to the physiology of patients. The Ethics Committee of First Hospital of Shanxi Medical University exempted patients from informed consent. We reviewed the imaging data of all patients with lung nodules who underwent ^18^F-FDG PET/CT scans between March and July of 2019 in our hospital. A total number of 75 Pulmonary nodules from 26 patients (20 males and 6 females; median age 66 years old, range 36–82 years old; median height 170 cm, range 155–183 cm; median weight 61.5 kg, range 40–100 kg) with a long-axis diameter of ≤ 25 mm on CT lung window were enrolled. The nodules sized under 10 mm were categorized as the sub-centimeter group, while the medium-size group was from 10 to 25 mm. The reconstructed PET images were processed using the PET Volume Computer-Assisted Reading (PET VCAR, GE Healthcare) on an Advantage Workstation 4.7 (GE Healthcare), which allows automatic segmentation based on the iterative adaptive algorithm^34^. We measured SUVmean and SUVmax of small pulmonary nodules.

### PVC for pulmonary nodules

A logarithmic model was generated based on the relationship of RC and sphere diameter (measured on CT images) in each reconstruction method. The partial volume effect correction was performed on all pulmonary nodules, based on the small-size nature, by dividing the corresponding RC. The PVC percentage increase, %ΔSUVmeans, was calculated as the difference between PVE corrected SUVmeans and original ones (4).

4 $$\mathrm{\%\Delta SUVmeans}=\frac{PVC SUVmeans-SUVmeans}{SUVmeans}\times 100\%$$

### Statistical analysis

Statistical analysis was conducted using SPSS Statistics 25.0 (IBM Co., New York, USA). Kruskal Wallis H test was utilized to analyze differences in phantom data (RC and CR) and in clinical data (SUVs) among the four reconstruction algorithms. Mann–Whitney U test was used to compare the SUVmean difference before and after PVC. P value < 0.05 was considered as statistically significant differences, while P < 0.001 was taken as highly significant differences.
